# Supplementary material for: Are carbon emissions trading and green financial instruments synergistic? -Comprehensive quantitative research based on content analysis
Source: PLoS One. 2024 Mar 7;19(3):e0298601. doi: 10.1371/journal.pone.0298601 (PMC10919601; doi:10.1371/journal.pone.0298601)
Supplement: S1 Data — (ZIP) [file pone.0298601.s001.zip › Data/地级市Z_F工作报告.zip!/╕╩╦α/╞╜┴╣╩╨/└┤╫╘╣1⁄2╓┌║┼í╛┬φ┐╦╩2╛▌═°í┐_2012.docx]

各位代表：

现在，我代表市人民政府向大会作工作报告，请予审议，并请政协委员和列席人员提出意见。

五年政府工作回顾

市二届人大一次会议以来的五年，是平凉发展史上极不平凡的五年。五年来，在市委的坚强正确领导下，在市人大、市政协的监督支持下，市政府团结带领全市各族人民，坚持以科学发展观为指导，认真落实全省区域发展战略和我市“四三二一”战略思路，努力克服国际金融危机和多种自然灾害的严重影响，抢抓机遇，锐意进取，集中突破，攻坚克难，全力推进新跨越、建设新平凉,全面完成了各项目标任务,经济社会发展取得了历史性成就。

过去五年，我们强力推进跨越发展，坚持不懈抓项目、争投资、扩总量，综合经济实力显著增强。紧扣发展第一要务，抢抓各类政策机遇，不断强化大项目、大产业、大企业和县域经济支撑，主要经济指标连续保持两位数增长，“十一五”规划提前一年完成。累计实施亿元以上各类项目166项，其中十大过10亿元工程建成8项，完成全社会固定资产投资1008亿元，是上一个五年的4.9倍。预计2011年全市地区生产总值完成272亿元，比2006年翻一番多，人均生产总值由5742元提高到13139元；大口径财政收入、地方财政收入完成35.8亿元和15.3亿元，分别增长1.7倍和1.6倍，财政收入占生产总值的比重提高到13.2%；财政支出完成89.1亿元，增长2.2倍。综合经济实力在全省排名上升到第六位。全市经济发展步入总量扩张、增速加快、质量效益快速提升的新阶段。

过去五年，我们着力转变发展方式，坚持不懈扩规模、抓延伸、打品牌，四大产业支撑效应显著提升。始终把做大做强主导产业作为调整经济结构、转变发展方式的着力点和突破口，持续推进煤电、草畜、果菜、旅游产业深度开发。能源综合开发进入国家区域发展战略重点，煤炭、石油资源勘探取得重大突破，新增煤炭探明储量44.1亿吨，煤矿、电厂、煤制甲醇等一批煤电化项目建成投产，煤炭产能、火电装机容量分别达到2500万吨和420万千瓦。新建、改造了一批农产品加工企业，扶持装备制造业持续做大，引进建成全省最大的水泥生产企业平凉海螺公司，以煤电化为引领，农产品加工、装备制造、新型建材、战略性新兴产业快速推进的新型工业化格局进一步巩固提升。2011年预计规模以上工业增加值完成80亿元，比2006年增长1.3倍，工业对财政的贡献率提高到64.3%。以牛、果为主的农村产业开发全面加快，肉牛饲养量、出栏量分别达到103万头和38.6万头；果树经济林达到185.7万亩，建成各类认证基地68.6万亩，果品产量达到89万吨，“平凉金果”、“平凉红牛”品牌效应日益扩大。大力推广以全膜双垄沟播为主的旱作农业新技术，粮食总产迈上百万吨台阶。累计投入23.4亿元，实施了一批景区开发和基础设施配套工程，崆峒山晋升为首批国家5A级旅游景区，成功创建“中国优秀旅游城市”，平凉旅游知名度和影响力不断扩大，以旅游为龙头的第三产业蓬勃发展。三次产业结构调整为20.59∶48.16∶31.25，四大产业集群优势日益凸显，在生产总值中的占比达到45%，全市形成了以工业为主导，以四大产业为支撑，一二三产协调推进的发展新格局。

过去五年，我们全力推动可持续发展，坚持不懈夯基础、破瓶颈、增后劲，发展条件显著改善。抢抓西部大开发、扩大内需和国家支持甘肃发展等重大机遇，集中实施了一批事关长远发展的重大基础设施、城镇扩容提质和生态建设项目。平定高速建成通车，西长凤高速、西平铁路、天平铁路建设加快，全市四级以上公路总里程突破6000公里，实现了乡乡通油路、58%的村通硬化路。建成了750千伏超高压输供电及一批农网、城网改造工程。实现了乡乡通光缆、50%的村通宽带。实施了50个乡镇（街道）公共视频监控系统建设，建成监控点位828个。累计投资189.1亿元，大力推进中心城市改造扩建和六县县城、重点小城镇建设，平凉城区基础设施配套进程加快，“六纵十横一环”路网框架基本形成。全市城镇化水平达到34.6%。新农村建设连片成带、区域化推进，以水路电气为主的农村基础设施建设加快推进，生产生活条件显著改善，近三成农户用上清洁能源。实施各类水利工程260处，新增有效灌溉面积3万亩，连续多年实现耕地占补平衡。完成造林封育102.9万亩，新修梯田104.6万亩，治理水土流失1100平方公里。以关闭“五小”为重点的环境治理成效明显，节能减排持续加强，全国生态示范区创建试点通过验收。全市呈现出不断强化基础、改善生态、拓展空间、提升要素聚集能力的良好态势。

过去五年，我们大力推进改革开放，坚持不懈抓招商、促创新、增活力，开放开发水平显著提高。坚持把改革开放、创新机制作为加快发展的强大动力，全面落实各项改革措施，全方位推进开放开发。完成了国有企业改革改制和市县（区）政府机构改革，积极实施了事业单位改革。财税征管、国有资产管理、金融体制改革不断深化，组建了非税收入征管机构和平凉市商业银行及一批村镇银行、小额贷款公司，综合治税机制和城乡金融服务体系不断完善。集体林权制度改革在全国创造了经验。医药卫生体制改革有序推进，乡村、社区卫生服务机构实行了基本药物零差率销售。创新完善走出去、引进来等招商引资机制，积极参加各类大型经贸活动，成功举办了全国乡企贸洽会等重大节会，先后与华能、中水电、酒钢、中电科、海螺、雨润等大企业集团、高等院校、科研院所和周边市州建立了战略合作关系，一批中央骨干企业、民营企业集团落户平凉，实现了合作开发由工业为主向一二三产全面拓展。五年实施各类招商项目547个，到位资金337亿元。以平凉金果为代表的出口贸易实现重大突破，进出口总值比2006年增长2.6倍。走出了以创优环境为基点，以优势产业为依托，以引强入平为抓手，多层次多领域开放开发的新路子。

过去五年，我们努力推进统筹发展，坚持不懈增投入、抓提升、促协调，各项社会事业显著进步。千方百计加大社会事业投入，强化基层基础，完善功能配套，提高公共服务水平。落实教育优先发展战略，累计投资22.5亿元，新建、改扩建学校732所、158.5万平方米，消除了D级危房；认真实施教育布局结构调整，义务教育经费保障机制全面建立，“普九”债务化解销零；组建了平凉职教集团，6所职教学校晋升为“国重”，教育教学质量稳步提高。建立了市生产力促进中心和一批工程技术研究中心，34项科技成果获得省科技进步奖，63件发明专利获国家授权。累计投入5.8亿元，建成了市医院综合楼，改扩建了县级综合医院、中医院和乡镇卫生院，建成社区卫生服务机构7个、标准化村卫生室565个，城乡医疗卫生服务能力有效提升。改造、新建了市文化活动中心、市图书馆、柳湖书院，建成乡镇综合文化站86个、乡村体育活动场所853个，实现了农家书屋和文化信息资源共享工程村级全覆盖，一批优秀文艺作品在全国、全省获奖，群众性文化活动和全民健身活动广泛开展。建成村村通工程6120处，广播、电视综合覆盖率分别达到97.7%和93.9%。提前一年实现了人口和计划生育省级优质服务市目标，人口自增率控制在5.48‰以内。双拥共建活动持续深入，我市荣获“全国双拥模范城”称号。统计、档案、气象、地震、人防、外事侨务、信息化、地方志、红十字、残疾人等事业取得新的进步。全市上下关注支持社会事业，加大投入、改善硬件、提升软件的良好局面进一步形成。

过去五年，我们竭力保障和改善民生，坚持不懈办实事、解难题、促和谐，人民生活水平显著提高。始终把增进人民福祉作为一切工作的出发点和落脚点，全面落实惠民政策，有效解决事关群众切身利益的突出问题。连续五年公开承诺措办惠民实事50件117项，全市财政用于民生的支出占到总支出的70%以上。千方百计扩大就业，累计安置高校毕业生1.8万人，解决了3352户零就业家庭的就业困难，发放就业小额担保贷款13亿元，新增城镇就业6.3万人。完成农民工技能培训16.7万人次，年均输转劳务45.1万人。实施了城镇居民基本医疗、城镇职工生育保险和村干部养老保险，实现了村干部报酬财政发放，开展了新型农村社会养老保险试点，在全省率先实现了新型农村合作医疗全覆盖，率先解决了集体工、长期临时工、“五七工”、“家属工”养老保险及破产企业退休人员医疗保险等历史遗留问题，城乡社会保障体系进一步完善。实施了企业退休人员养老金提标、城乡低保扩面提标和农村五保供养提标，开展城乡医疗救助33.7万人次。建成了市儿童福利院、市流浪未成年人救助保护中心和一批养老福利机构。地震、洪涝灾后恢复重建任务全面完成。建成廉租房1.18万套，实施了城区棚户区和农村危旧房改造，有5384户群众搬出了土窑洞和地坑庄子。解决了73.9万人饮水安全问题，全市农村饮水安全覆盖率、自来水入户率分别达到92.1%和82.3%。加大连片开发扶贫力度，减少农村贫困人口21.6万人。全面推行惠农资金“一册明、一折统”支付制度，累计兑付各类政策性补贴53.7亿元。农民人均纯收入、城镇居民人均可支配收入分别比2006年增长85.9%和67.9%。全市进一步形成了高度关注民生、全力改善民生、提升共建共享水平的良好导向。

过去五年，我们紧紧围绕建设为民、务实、清廉政府，加快推进政府职能转变和管理创新，制定完善了政府议事规则和重大事项专家咨询、社会听证、新闻发布等制度，科学、民主决策水平不断提高。坚持依法行政，认真执行人大及其常委会的决议决定，自觉接受人大、政协和社会监督，共办理人大议案、代表建议353件，政协委员提案564件，办复率均达到100%。全面推行政务公开，加快电子政务建设，规范行政许可事项1042项，取消行政审批事项45项。深入开展学习实践科学发展观、解放思想、创先争优和机关作风整顿等活动，公务员队伍整体素质不断提升。认真落实党风廉政建设责任制，强化行政效能监察和责任追究，加强审计、财政监督，持续开展纠风治乱和“小金库”专项治理，政府系统廉政建设扎实推进。不断强化市场价格监管，市场秩序进一步规范。集中开展食品药品专项整治，完善机制，推行了食品安全城乡一体化监管模式。健全完善应急管理体系，及时有效应对“5·12”地震、“7·23”暴洪等自然灾害，快速处置了“问题奶粉”、甲型流感等突发公共事件。扎实推进平安建设，完善治安防控体系，依法严厉打击各类违法犯罪活动，深入排查调处矛盾纠纷，妥善解决信访突出问题，加强安全生产监管，依法管理宗教事务，加快发展民族乡村经济社会事业。全市民族团结、宗教和顺、安定和谐的良好局面进一步巩固发展。

即将过去的2011年，全市上下认真贯彻市二届十六次全委会暨全市经济工作会议精神，紧紧围绕市二届人大六次会议确定的目标任务，集中精力抓落实、求突破，经济社会发展实现了“十二五”良好开局。重大项目快速推进。省市列57个重点项目进展良好，光伏产业园一期投入试生产，碳纤维产业园加快建设，酒钢集团平凉煤电化循环经济项目启动实施，西长凤高速、静庄二级公路年底建成通车，平凉机场、铁路、公路等一批重大项目前期工作积极推进。全市固定资产投资预计完成325亿元，拉动经济增长8.7个百分点。能源化工基地建设进程加快。编制完成了能源化工基地发展《规划》，确立了5个项目功能区及开发重点，邵寨煤矿建设步伐加快，平凉电厂三期和华亭、崇信电厂二期、60万吨煤基烯烃、20万吨聚丙烯项目前期进展顺利，原煤产量、发电量分别达到2400万吨和170亿千瓦时。农村主导产业规模效益持续提升。新建规范化肉牛养殖小区51个，新植果树经济林21万亩，建成蔬菜生产集中区15个，蔬菜产量突破百万吨，主导产业占农民人均纯收入的比重达到46%。旅游商贸活力日益增强。积极开展了“神奇崆峒·养生平凉”品牌推介，实施了崆峒山景前区等一批旅游重点项目，成功举办了崆峒旅游文化节暨中华武林大会，预计游客接待量突破500万人次，综合收入超过20亿元。崆峒古镇、世博伟业建材家居广场投入运营。预计全社会消费品零售总额达到102亿元，第三产业增加值达到85亿元。对外交流合作不断深化。积极争取，多方努力，全市整体融入关天经济区、陕甘宁革命老区、蒙陕甘宁“金三角”能源综合开发等3个国家级发展规划。与11户央企签订了战略合作协议，引进台湾奥斯特公司投资开发红牛产业，“兰洽会” 项目签约总额位居全省前列，全市招商引资到位资金比上年增长25%。城镇建设步伐加快。编制各类规划186项，中心城市控制性详规实现全覆盖。新区绿地公园建设加快，南环路改造全面完成，312国道过境段改线全线贯通，崆峒大道改造提升、“八沟一河”综合治理等工程进展良好，六县县城和36个重点小城镇建设取得了新的进展。社会事业取得新进步。“两基”工作顺利通过国家验收，市特教学校完成迁建，平凉技师学院挂牌招生。市妇幼保健院新建工程基本竣工，市社会福利院、市精神病院建设进展良好。实施了市级广播电视节目无线覆盖工程，市广电新闻中心年内开工建设。成功举办了市第三届运动会，市体育中心前期工作加快推进。民生保障力度加大。省市确定的实事全面完成。各类保障性住房开工率、主体竣工率分别达到100%和60%，基本解决了城镇人均13平方米以下家庭的住房困难。城镇居民养老保险和新农保国家试点扩大到5个县（区）。新增城镇就业2.7万人，城镇登记失业率控制在4%以内。

各位代表，市二届人大一次会议以来的五年，是我市产业发展最为迅猛、经济实力增长最快的五年，是基础设施和生态环境建设投入力度最大、成效最为显著的五年，也是各项社会事业发展最好、城乡面貌变化最大、人民群众得到实惠最多的五年。这些成绩的取得，是市委坚强正确领导的结果，是全市各族人民团结奋斗的结果，是社会各界大力支持的结果。在此，我代表市人民政府，向辛勤工作在各条战线的广大干部群众，向给予政府工作大力支持的人大代表、政协委员、各民主党派、工商联、无党派人士和各人民团体，向中省驻平单位和驻平部队、武警官兵，向为平凉发展作出贡献的老同志，向关心、支持平凉改革发展的各界人士，表示衷心的感谢并致以崇高的敬意！

五年的砥砺奋进，取得了巨大成就；五年的伟大实践，积累了宝贵经验。我们深深体会到，推动全市经济社会跨越发展，一定要紧紧抓住第一要务，千方百计加快发展步伐，着力做大经济总量，不断壮大经济实力；一定要加快经济结构调整和发展方式转变，坚持好中求快、快中求好，着力提升发展质量和效益；一定要抓基层、打基础、利长远、增后劲，在攻坚克难中全力打造后发优势；一定要不断深化改革，扩大开放，创新招商引资机制，大中小齐抓，内外商并重，在创优环境中努力提升区域竞争力；一定要坚持大区域布局、大项目支撑、大工程引领，持续强化投资拉动，多层次多领域培育新的经济增长极；一定要加快培育主导产业，推进优化升级，集约化、集群化发展，大力提升主导产业支撑能力；一定要坚持以人为本，突出普惠性，注重实效性，持续改善民生，让人民群众共享改革发展成果；一定要求真务实，崇尚实干，全力以赴抓落实、干到位，以一流的作风保障和促进跨越发展。

在总结经验发扬成绩的同时，我们也清醒地认识到，政府工作和全市经济社会发展中还存在不少困难和问题：经济总量小，人均水平低，自主创新能力不强，调整经济结构和转变发展方式任务艰巨；农业基础比较薄弱，特色产业链条短，农民持续增收的难度较大；基础条件瓶颈制约因素还比较多，生态环境仍然比较脆弱，节能降耗和环境约束压力加大；民营经济发展缓慢，就业形势依然严峻；民生保障层次低，部分低收入群众生活还比较困难，公共产品供给能力和公共服务水平与人民群众的愿望仍有较大差距；少数干部思想观念、精神状态和工作作风还不能适应跨越发展的要求，等等。我们将采取有力措施，认真解决，努力把全市经济社会发展不断推向新阶段。

未来五年政府工作和2012年主要任务

未来五年，是我市全面建设小康社会的关键五年，也是快转型、促跨越、大发展的战略机遇期、蓄势腾飞期和攻坚破难期。我们面临的宏观经济环境更加复杂、要素制约矛盾更加突出、区域竞争更趋激烈，但总体上机遇大于挑战。国家支持西部、支持甘肃的一系列政策措施全面落实，我市整体融入国家层面的战略规划启动实施，全省区域发展战略深入推进，为我市率先发展、加快跨越提供了难得的历史机遇和政策支持。刚刚召开的市第三次党代会对全市今后发展作出了全面部署，确立了“加快转型跨越，促进科学发展，建设小康平凉、和谐平凉、文明平凉、生态平凉”的总目标、总要求，进一步统一了广大干部群众的思想，提振了信心，凝聚了力量，必将激励全市人民积极投身推动转型跨越的伟大实践，努力创造更加辉煌的成就。

政府工作的总体要求是：高举中国特色社会主义伟大旗帜，以邓小平理论和“三个代表”重要思想为指导，深入贯彻落实科学发展观，以科学发展为主题，以加快转变经济发展方式为主线，以显著提高人民生活水平为目的，按照省委省政府跨越式发展的总要求，全面落实“四三二一”战略思路，加快转型跨越，促进科学发展，努力建设小康平凉、和谐平凉、文明平凉、生态平凉，为全面建成小康社会打下坚实基础。

转型跨越是实现科学发展的必然选择，建设小康和谐文明生态平凉是全市人民的共同愿望。在新的起点上，我们必须紧紧抓住事关经济社会发展的重大问题，集中各方面智慧和力量，强化统筹协调，全力攻坚破难，奋力开创全市科学发展新局面。

与“十二五”规划相结合，未来五年政府工作的战略重点是：

（一）加快产业升级，优化经济结构，着力构筑转型跨越的重要支撑。持续做强优势产业，改造提升传统产业，加快培育新兴产业，打造特色品牌，构建结构合理、优势突出、关联度高、竞争力强、支撑作用明显的现代产业体系。大力实施工业强市战略，加快新型工业化进程。突出陇东能源化工基地建设，加大煤炭石油资源勘探开发力度，加快实施酒钢150万吨低镍生铁等3个投资过百亿元项目、平凉电厂三期等6个投资过50亿元项目、光伏产业园等8个投资过10亿元项目，促进煤电化、煤电冶、煤电材一体化发展；强化外引内联，着力构建农产品精深加工体系；加快出城入园，推动阀管刀和矿用机械、石油机械配件等装备制造业改造升级、集团化发展；依托峡门集中区，持续做大做强新型建材业；突出光伏、碳纤维、电石、PVC、电子信息、生物医药等重点，加快培育新兴产业集群。集中建设绿色畜牧、优质果品基地和干旱山塬区现代农业示范区，提升农业产业化水平。加快实施“四个百万” 工程（粮食产量稳定在100万吨以上、肉牛饲养量达到120万头以上、果品总产量达到110万吨以上、蔬菜面积达到100万亩以上），大力推进草畜、果菜产业规模化、标准化、集约化、品牌化开发，构建起支撑力强的现代农业产业体系。全面推进文化、旅游产业相互融合，迅速壮大第三产业。深度挖掘地域特色文化内涵，实施一批重大文化旅游产业项目，培育一批辐射带动力强的文化旅游骨干企业，全方位打造“神奇崆峒·养生平凉”旅游品牌，着力提升以崆峒山旅游区为中心，以泾川旅游区、关山休闲避暑旅游区、灵台文化旅游区、葫芦河农业生态观光旅游区为基点的文化旅游综合开发水平，争创中国最佳旅游城市，推动以文化旅游为龙头、现代服务业为主体的第三产业快速发展。

（二）突出城镇带动，推进新农村建设，着力强化转型跨越的聚集功能。坚持统筹城乡产业布局、基础设施建设和社会事业发展，全面提升中心城市、六县县城、重点城镇、中心村镇的要素聚集能力。大力实施中心城市带动战略。按照建设大景区、大园区的思路，坚持规划一张图、建设一盘棋、管理一体化、服务一条龙，进一步健全完善建管分离、重心下移、责权明确、市区一体化管理机制。全面完成热电联产集中供热和污水垃圾处理项目，同步配套建设地下综合管网。加快建设中心商务区、工业集中区、生态居住区和文体功能区。实施天门塬开发及一批基础设施、生态景观、地标建筑等扩容性、功能性、提质性工程，中心城市人口达到42万人，建成西部聚集辐射效应明显的区域性中心城市。着力构建功能完善的城镇体系。持续推进六县县城和重点小城镇功能配套。加快公共资源向村镇延伸，引导农民向城镇和非农产业有序转移。全市城镇化水平提高到39%以上。全面加快新农村建设。坚持新村建设与旧村改造相结合，基础设施与产业配套相结合，扶贫开发与小康建设相结合，以中心村建设为重点，特色化、区域化推进，促进农村资源要素快速聚合。2016年全市新农村建设和“三清五改”示范村覆盖面分别达到45%和85%，基本消除农村危房。

（三）建设“三大枢纽”，强化基础设施，着力改善转型跨越的基础条件。以交通、电力、水利为重点，突破瓶颈制约，改善硬件条件，增强发展后劲。争取实施宝中二线等5条铁路、平天等6条高速公路和平凉机场建设项目，改造县乡公路1000公里，硬化通村公路4000公里，力争县县通高速公路、乡镇通等级公路、建制村通硬化路，加快构建交通枢纽。规划建设±800千伏直流、1000千伏交流特高压输供电工程，扩建750千伏超高压变电站，新建一批330千伏、110千伏送变电工程，形成西电东送枢纽。依托铁路、公路、园区，建设一批大型专业市场和仓储设施，形成西部重要的煤化、农副产品、新型建材、新材料集中生产区、储备区和集散地，加快构建物流枢纽。加强信息化建设，推进“三网”融合，打造“数字平凉”。强化水利建设，建成一批水源调蓄调配、水库除险加固、灌区改造、饮水安全、梯田综合开发、山洪灾害防治工程，建成自来水化市、梯田化市。

（四）加强生态建设，发展循环经济，着力提升转型跨越的承载能力。以建设国家生态示范市、全国园林城市、全省循环经济示范市为统揽，持续加强生态建设和环境保护，强化节能减排，集约节约利用资源，增强可持续发展能力。抓好林业重点生态工程建设，集中实施一批整流域、整区域山水林田路综合治理和生态修复工程，发展生态经济，建设生态城市，促进生态环境持续好转，全市森林覆盖率达到32%以上。认真落实循环经济发展规划，按照减量化、再利用、资源化要求，加快实施酒钢平凉煤电化循环经济项目，集中实施一批工业、农业循环工程，推广清洁生产，倡导低碳、绿色生活，提高资源利用效率。持续抓好重点行业、重点企业和社会节能，实施一批节能减排技改项目，有效提升市县两级污水、垃圾处理能力，建立健全重点污染源监控体系和环境综合治理齐抓共管机制，全面治理大气污染、水污染、矿区污染和农村面源污染，生态、循环、节能、减排综合推进，不断扩大环境容量。

（五）推进改革创新，扩大交流合作，着力增强转型跨越的动力活力。拓宽思路，积极探索，深化改革，创新机制，进一步促进开放开发，充分调动各方面积极性、创造性，激发干事创业、投资兴业的热情。实施人才强市战略，建立健全人才培养引进、使用管理机制。抓好财税改革，加强综合治税，健全公共财政体系，探索建立新的财政管理体制下市区财政保障新机制。深化金融改革，构建多元化融资平台。积极稳妥推进土地承包经营权流转，进一步深化水利、集体林权、国有林场改革。稳步推进事业单位改革，加快文化、教育、科技、医药卫生体制改革，促进公共资源均衡配置。全方位、宽领域、高层次推动交流合作，加快引强入平，全面提升招商引资规模和质量。继续深化与央企、发达地区和周边城市的合作、协作，主动融入关天经济区，优化投资环境，突出项目招商、产业融合，积极对接大基地、大企业、大园区，发展一批协作配套、相互支撑、吸纳就业能力强的企业集群。落实各项扶持政策，加强服务平台建设，促进民营经济健康快速发展。

（六）加强社会建设，改善民计民生，着力营造转型跨越的和谐环境。下功夫解决群众最关心、最直接、最现实的利益问题，全面推进社会事业发展，加强社会建设和管理，构筑多层次、广覆盖、高质量的公共服务体系，不断夯实和谐基础。深入贯彻党的十七届六中全会精神，加快建设文化强市。大力发展公益性文化事业，加大市县图书馆、文化馆、博物馆、科技馆以及广电、体育中心等文化基础设施建设力度，加强文化人才队伍建设；加快发展文化产业，深度挖掘崆峒山、西王母、皇甫谧、古成纪等地域特色文化内涵，保护整理文化遗产，建设优秀传统文化传承体系；深入推进文化科技创新，促进文化与相关产业融合，加快建设文化产业园，创作推出一批文艺精品，扩大文化消费，构建文化产业体系，努力开创以提高全民科学文化素质为目的，基础设施、人才队伍、文化产品三位一体推进的文化发展新局面。加快调整优化教育布局结构，积极创办教育集中区，持续改善办学条件，逐步实现义务教育均衡化、学前和高中教育普及化、职业教育大众化。加大医疗卫生项目实施力度，强化社区卫生服务能力，扶持发展中医药事业，合理配置医疗卫生资源，不断完善城乡医疗和公共卫生服务体系。认真落实全民科学素质行动计划《纲要》，加大科技创新和实用技术推广力度，加快科技成果产业化进程。加强人口和计划生育工作，稳定低生育水平。大力弘扬“包容、和谐、务实、进取”的平凉精神，广泛开展各类文明创建活动，着力提高全民素质和城乡文明程度。加强就业培训服务体系建设，鼓励支持以创业促就业，千方百计扩大就业。加快建立覆盖城乡的社会保障体系，努力实现城乡居民社会养老保险、城镇基本医疗保险全覆盖。健全以城乡最低生活保障、五保供养、大病医疗救助、特困群众救济、残疾人保障为主的新型社会救助体系，积极发展社会福利和养老事业。继续坚持为民措办实事制度。抓住进入六盘山扶贫片区机遇，加大整村推进连片开发扶贫力度，贫困面下降到10%以内。持续推进社会管理创新，健全科学有效的利益协调机制、诉求表达机制、矛盾调处机制和权利保障机制。扎实推进“六五”普法，积极开展法律援助，加快依法治市进程。深入开展双拥共建，扶持民族乡村加快发展，依法加强宗教事务管理,不断增进民族团结、军政军民团结，全面推进社会和谐进步。

2012年，是全面加快转型跨越的起步之年。全市经济社会发展的主要预期目标是：地区生产总值增长14%；规模以上工业增加值增长16%；固定资产投资增长15%；地方财政收入增长15%；社会消费品零售总额增长16%；城镇居民人均可支配收入增长12%；农民人均纯收入增长15.6%；城镇登记失业率控制在4%以内；人口自增率控制在7.1‰以内。

2012年的主要任务是：

（一）加快推进转型升级，大力提升工业经济质量效益。坚持扩张总量和优化结构并重，着力延伸产业链条，壮大工业集群。加快能源综合开发。完成灵台独店—泾川高平煤炭资源精查和矿井规划设计，开展泾川东部、崆峒区北部煤炭资源地质调查，争取开建灵台唐家河、南川河和崇信赤城、五举煤矿，加快邵寨煤矿建设进度，确保大柳、新安煤矿达产达标，百贯沟、华星公司60万吨矿井投入试生产。开工建设中煦20万吨聚丙烯项目，加快平凉电厂三期和华亭、崇信电厂二期以及240万吨兰炭项目前期工作。原煤产量达到2600万吨，发电量190亿千瓦时。促进装备制造业扩容增效。抓好红峰减温减压疏水阀研发生产、虹光真空接触器和高频无极荧光灯生产线建设，确保亨达印机改造提升项目达产达标，完成丰收公司并购重组和出城入园，力争临工机械制造项目开工建设。积极发展新兴产业。全面建成光伏产业园一期和碳纤维复合材料产业园一期项目，开工建设2.8兆瓦太阳能光伏发电项目。开发省级新产品10项以上。改造升级农产品加工业。加快天纤棉业、皇甫谧制药、恒达废纸综合利用等重点项目进度，新建通达果渣综合利用生产线，扩大静宁纸箱包装产业园规模，改扩建30户龙头企业，支持果畜等加工企业做大做强。提升新型建材业发展水平。海螺水泥总体实现达产达标，推进祁连山日产2×4500吨新型干法水泥项目前期工作，加快培育一批煤矸石制砖、粉煤灰综合利用、新型墙体材料等新型建材企业。加强工业园区（集中区）建设。积极探索完善管理模式，合理规划功能分区，大力度推进以路水电讯为重点的基础设施建设，确保新入园千万元以上项目20项。申报国家级经济技术开发区取得实质性进展。

（二）加快推进特色产业提质增效，全力促进农村经济快速发展。突出产业化、集约化，着力提高农产品附加值和竞争力，认真落实各项强农惠农政策，多环节多渠道增加农民收入。持续推进牛果菜产业开发。加强基础母牛保护，推进秸秆转化利用，新建肉牛标准化养殖小区20个，加强与奥斯特集团合作，完成红牛大厦和红牛产业园建设前期工作；新植果树经济林25万亩，巩固提升标准化生产基地114万亩，果品产量达到92万吨，优果率提高到65%；新增设施蔬菜5000亩，蔬菜总产量达到100万吨以上。稳定提高粮食综合生产能力。完成全膜双垄沟播种植148万亩、全膜覆土穴播小麦30万亩，推广测土配方施肥430万亩，粮食总产稳定在100万吨以上。完善农业产业化服务体系。突出抓好农技推广、疫病防控、质量监管、农产品销售及信息服务等，加快农业机械化进程，创建2个省级农业示范园区，发展农村专业合作经济组织50个。积极发展劳务经济。坚持就地就近转移与有组织输出相结合，实施农民工职业技能培训2万人，输转劳务47万人，创劳务收入45亿元以上。扎实推进新农村建设和扶贫开发。继续抓好省列新农村试点县和3个城乡一体化试点乡镇，抓建新农村建设试点村20个、“三清五改”示范村100个。实施整村推进项目35个，抓好华亭县黑河流域省列片区开发和六盘山特殊贫困片区5个整乡推进试点，完成易地扶贫搬迁1145户5788人，减少贫困人口3万人。全市农业增加值增长6.5%。

（三）加快推进旅游产业开发，有效提升现代服务业水平。深度开发以“神奇崆峒·养生平凉”为主题、以特色文化为内涵、以精品景区为支撑的旅游产业，抓好平泾路崆峒山连接线、大云寺三期及云崖寺景区配套提升等9个重点项目，新建中国梯田化第一县博物馆，加快界石铺红军长征纪念园、莲花台国家4A级景区创建步伐，整顿规范农家乐，提升崆峒古镇经营管理水平，全力办好第六届世界养生大会暨第二届中华武林大会，着力开发旅游系列产品，全方位开展宣传推介，开拓中远程客源市场。全年旅游接待人数达到630万人次以上，旅游综合收入达到28.5亿元。开工建设平凉工业园区综合物流中心，抓好陇东农产品批发交易中心、风尚吉伍国际建材、新天地购物广场等商贸设施和专业市场建设，实施酒钢生活基地项目。继续推进退市还路、万村千乡市场工程，新建一批农村连锁超市以及城区便民市场和“放心粮店”。大力发展金融保险、仓储配送、电子商务等生产性服务业，壮大提升住宿餐饮、家政服务、文化娱乐等生活性服务业，促进房地产业健康发展。提升出口能力，进出口总值增长20%。全市第三产业增加值增长13.5%。

（四）加快推进基础设施建设，持续改善城乡发展条件。建成西平铁路、天平铁路和泾渗、韩店至张家川二级公路，实施平泾、崇白公路改建工程，力争平凉机场批复立项，加快推进平庆铁路及惠宝、平天、静庄、华崇灵高速前期工作。新建平凉客运枢纽站、快客客运站和市道路运输应急救援指挥中心。实施各类水利工程60项，完成10座水库除险加固，加快农田水利建设和中小河流域治理，推进引洮工程静宁受水区项目、兔里坪补偿水库前期工作，建成一批灌区续建配套与节水改造工程，新增有效灌溉面积5000亩。加快建设2个330千伏和3个110千伏送变电工程，抓好农网改造升级，提升电力保障能力。实施城市光纤到户和农村光缆进村、宽带入户工程，提升信息化建设和应用水平。

（五）加快推进城镇化建设，不断提升辐射带动能力。完成第四轮中心城市总体规划修编，高标准编制重要节点、重点功能区修建性详规。县城控制性详规覆盖率和村庄规划制定率达到100%。突出中心城市功能配套，组建市投资开发公司，搭建中心城市建设融资平台；完成312国道过境段改线工程和双桥路泾河大桥主体，全面建成新区绿地公园；实施柳湖路东拓、双桥路北拓和东大门道路、平沿路改造；大力推进棚户区和城中村改造，加大保障性安居工程建设力度；抓好“八沟一河”综合治理、天雨污水处理厂升级改造等工程，扩建生活垃圾处理场，新建建筑垃圾处理场，推进城区绿化亮化和柳湖公园、火车站广场、崆峒大道西段、解放北路等景观提升工程。集中实施一批县城、重点小城镇基础设施和功能配套项目，积极推进中心村建设和村庄环境综合整治。加强城市管理，严格规划执法，建设综合数字城管系统，集中开展市容市貌综合整治和文明市场、文明街道、文明社区、文明工地创建活动，规范户外广告和小区物业管理，实现综合执法与日常管理向背街小巷、居民社区、城乡结合部延伸，全面提升城市文明形象。

（六）加快推进生态建设，切实强化节能减排。持续抓好三北、天保等重点工程和城镇面山、道路绿化，实施泾渭河流域百万亩生态防护林基地建设和亚行贷款项目，完成造林封育16万亩。抓好巩固退耕还林口粮田工程和坡耕地水土流失综合整治试点，新修梯田22万亩，治理水土流失220平方公里。完成土地开发整理复垦任务，新增耕地8000亩。严格饮用水水源地保护和地下水采用管理，促进全社会节约用水。加强矿产资源开发监管，落实矿山环境恢复治理保证金制度。实施20个重点节能技改项目，抓建8户省级循环经济示范企业，深入开展公共机构和社会节能，万元生产总值能耗控制在目标值以内。严格项目准入，抓好重点行业、企业废水治理和循环利用工程建设，完成电厂、水泥等企业烟气脱硫脱硝工程，开建3个县城污水处理项目，实施38个农村环境集中连片整治和19个规模化养殖场污染治理项目，确保完成主要污染物减排约束指标。

（七）加快推进改革开放，努力扩大招商引资规模。认真做好分离企业办社会职能工作。全面推行事业单位公开招聘制度。稳步推进经营性文化单位改革。积极探索公立医院改革，加快基本药物制度全覆盖进程。扎实推进林权制度配套改革。探索建立农户自愿退出土地承包经营权和宅基地使用权补偿机制。扩大政策性农业保险试点范围。进一步完善机制，多形式搭建平台，多领域深化合作，强化签约项目跟踪服务，全面提高履约率，全年实施招商引资项目100项，到位资金125亿元以上。实施“50强民营企业”创业帮扶工程，加大对中小企业的金融支持力度，大力发展中小企业集群。

（八）加快推进社会事业发展，全面提升公共服务水平。认真落实市委关于学习贯彻党的十七届六中全会《决议》精神，推动文化事业、文化产业加快发展。加大投入力度，启动实施以市博物馆二期、文化馆等为重点的文化基础设施项目；加强基层文化阵地建设，完善公共文化服务网络，深入开展“平凉文化年”系列活动，推动文化科技卫生“三下乡”、“进社区”等经常化；整合文化资源，充实力量，强化保障，全面推进市县（区）“三馆”、乡镇文化站免费开放，着力提高现有文化资源利用效率，不断活跃城乡群众文化生活；坚持引进与培训相结合，加强城乡一体的文化人才队伍建设；扎实开展优秀文化遗产保护开发，加大文艺精品创作力度，力争文化产业园立项建设，打造具有地域特色的文化产业发展新优势。持续推进中小学校布局调整和标准化建设，新建、改建校舍15.9万平方米，完成平师附小改扩建，建成泾川特教学校；加快平凉医专升本步伐，力争平凉职业技术学院挂牌招生并启动新校区建设；推进职业教育集团化办学试点；实施农村义务教育学生营养改善计划；加强教师队伍建设管理，推进素质教育，提高教育教学水平。巩固完善新型农村合作医疗制度，建成市精神病院综合楼，确保5个县级医院、7个中心乡镇卫生院改扩建项目建成投用，新建30个标准化村卫生室。加快市科技企业孵化器暨生产力促进中心综合楼建设步伐。建成市广电新闻中心主体，实施省级广播电视节目无线覆盖工程。建设全国人口和计划生育利益导向政策体系示范区，提高优质服务能力。

（九）加快推进社会管理创新，进一步夯实社会和谐稳定基础。积极探索以社区为重点、强化基层管理促进社会管理的新机制，合理调整城市社区规模，适当增加社区数量，充实工作力量，完善基础设施和服务功能，努力实现管理的全员覆盖、全域覆盖。突出社会面稳控和治安防控体系建设，加强流动人口、重点人群、重点区域管理，强化信息网络监管，提高对虚拟社会的管理水平，严厉打击各类违法犯罪活动。强化信访工作责任制和领导包案制，着力构建信访、司法、法制、综治、民调“五位一体”联动调解机制，拓宽社情民意表达渠道，及时解决群众合理诉求。严格安全生产责任，推进安全标准化建设，集中开展打非治违专项行动，促进安全生产形势持续好转。整顿规范市场秩序，完善价格补贴和重要商品储备制度，保障市场供给，稳定价格水平。扎实推进食品药品安全监管城乡一体化建设，积极创建国家食品药品安全示范县（区），切实保障人民群众饮食用药安全。建成市救灾物资储备库，健全自然灾害预测预警机制，进一步完善防灾减灾体系。加强应急管理体系建设，切实提高应对突发公共事件的能力。

（十）加快推进民生工程，集中办好惠民实事。强化就业服务和就业援助，着力解决普通高校毕业生和城镇新增劳动力、就业困难人员就业问题。加强劳动保护，切实维护农民工合法权益。进一步扩大社会保障覆盖面，新农保和城镇居民养老保险参保率分别达到90%和80%以上。建成市级人力资源市场和社会保障服务中心。加强城乡低保规范化管理，积极开展社区养老服务试点。统筹抓好城乡医疗救助、五保供养、救灾救济工作。同时，集中办好十件惠民实事：1．新增城镇就业2.7万人，80%以上普通高校应届毕业生实现就业。2．新建各类保障性住房3000套以上，实施农村危旧房改造2万户，完成“告别窑洞”工程。3．新建集中供水工程16处，解决15万人饮水安全问题，基本实现农村饮水安全全覆盖，全市自来水通村率达到100%。4．实施县乡道路改造100公里，新建通村硬化路450公里，开通城乡公交班线12条。5．开工建设平凉城区热电联产集中供热工程，同步实施雨水、污水全收集改造；更新公交车25辆，新建公厕10座，完成路口交通信号灯配备。6．新建、改扩建公办幼儿园15所。7．建设市全科医生临床培养基地和市级儿童专科病区；建成市、县、乡三级“120”急救调度指挥系统，为乡镇卫生院配备急救车及车载设备，实现“120”城乡全覆盖。8．建成6个乡镇体育惠民工程；完成农村广播“村村响”工程，建设2座城市数字影院。9．实施40个乡视频监控系统建设，建成公共区域视频监控点位2635个。10．建成“一池三改”沼气生态农户1万户、村级服务网点50个；为5000户农户配发太阳灶。

加强政府自身建设

面对发展的重任、人民的期盼，我们将按照行为规范、运转协调、公正透明、廉洁高效的要求，进一步转变政府职能，推进管理创新，提升服务效能，把加快转型跨越、促进科学发展的各项任务目标全面落到实处。

深入推进思想解放，不断激发创新活力。大力推进学习型机关建设，全面提高各级公务人员的思想觉悟、理论素养、政策水平、精神境界，在服务转型跨越的具体实践中，以强烈的大局意识、责任意识、奉献意识，勤奋敬业，敢于负责，勇于担当；以新的理念、新的思维，勇挑重担，攻坚克难，创造性开展工作；以昂扬向上、奋发有为的精神状态，积极进取，干事创业，争创一流，促使各级公务人员始终做到以人民利益为重、以平凉发展为重，形成人人干事有激情、个个单位有活力、凝心聚力抓落实的良好局面。

深入推进依法行政，不断提升政府公信力。全面推进法治政府建设。进一步完善政府议事规则及重大决策咨询、听证、公示、风险评估等制度，提高决策的科学化、民主化、规范化水平。严格落实行政执法主体责任制和执法过错追究制，细化量化行政裁量权，进一步规范各级行政机关施政行为，做到依法用权、秉公用权、为民用权。主动接受人大法律监督、政协民主监督和社会舆论监督，认真落实人大及其常委会决议决定，及时办理人大代表建议和政协委员提案。坚持以有为干事促进跨越，以诚信干事服务人民，做到决策务必落实、承诺坚决兑现。加大与人民群众息息相关的办事程序、收费标准、补贴政策、规定依据等公开力度，深入推进电子政务建设，不断完善新闻发布制度，切实保障人民群众的参与权、知情权和监督权。以各级公务人员的依法办事、诚信干事、高效干事，凝聚人心，团结群众，齐心协力谋发展、促跨越。

深入推进职能转变，不断提升公共服务能力。进一步理顺部门职能，加快政府职能向创设良好环境、提供优质服务、维护社会公平正义转变。优化公共资源配置，加大财政对民生、“三农”、社会事业和公共设施的投入力度，努力为人民群众提供更多的优质公共产品。进一步清理规范行政审批和行政许可事项，简化办事程序，严格执行首问负责、限时办结、公开承诺、责任追究制度，下决心整治和解决一些部门和单位推诿扯皮、效率不高以及部门利益至上、不作为、乱作为等突出问题，建立责权一致、分工合理、规范有序、务实高效的运行机制，真正做到在其位谋其政、履其职尽其责，切实提高服务质量和效率。

深入推进作风建设，不断提升政府执行力。坚持问政于民、问计于民、问需于民，始终以群众呼声为第一信号，以群众需要为第一选择，以群众满意为第一标准，大兴调查研究之风，下移工作重心，全面推行一线工作法，集中精力抓落实、干实事、求实效。进一步健全完善目标责任管理和绩效考核机制，对重点工作、重大项目和民生实事，责任到领导、到部门，严格时间进度和质量标准，及时督查考核，奖惩兑现，做到令必行、行必果，在政府系统进一步营造务实干事、立决立行、落实有力、一抓到底的浓厚氛围。

深入推进反腐倡廉，不断增强拒腐防变能力。严格落实党风廉政建设责任制和廉洁自律各项规定，健全惩治和预防腐败体系，全面加强政府系统党风廉政建设。强化对工程招投标、资源开发以及土地出让、产权交易等领域的监管，从源头上预防和治理腐败。持续开展民主评议政风行风和软环境“双评”活动，坚决纠正部门和行业不正之风。强化行政监察和审计监督，进一步完善重大工程、重大项目和专项资金监督检查机制，严肃查处各类违法违纪案件。深入推进节约型机关建设，严控“三公”支出，杜绝铺张浪费，切实降低行政成本。加强公务人员廉政教育，不断提高自我规范、自我约束、清廉从政的自觉性，以廉政取信于民，以勤政造福于民，进一步树立和维护政府的良好形象。

各位代表，加快转型跨越，促进科学发展，已成为全市上下统一的意志和行动。让我们在市委的坚强领导下，团结奋进，锐意创新，扎实苦干，顽强拼搏，为实现小康平凉和谐平凉文明平凉生态平凉的宏伟目标而努力奋斗！
